# Application of Celluspots peptide arrays for the analysis of the binding specificity of epigenetic reading domains to modified histone tails

**DOI:** 10.1186/1471-2091-12-48

**Published:** 2011-08-31

**Authors:** Ina Bock, Srikanth Kudithipudi, Raluca Tamas, Goran Kungulovski, Arunkumar Dhayalan, Albert Jeltsch

**Affiliations:** 1 Biochemistry Laboratory, School of Engineering and Science, Jacobs University Bremen, Campus Ring 1, 28759 Bremen, Germany; 2 Department of Biotechnology, Pondicherry University, R.V. Nagar, Kalapet, Puducherry - 605014, India

## Abstract

**Background:**

Epigenetic reading domains are involved in the regulation of gene expression and chromatin state by interacting with histones in a post-translational modification specific manner. A detailed knowledge of the target modifications of reading domains, including enhancing and inhibiting secondary modifications, will lead to a better understanding of the biological signaling processes mediated by reading domains.

**Results:**

We describe the application of Celluspots peptide arrays which contain 384 histone peptides carrying 59 post translational modifications in different combinations as an inexpensive, reliable and fast method for initial screening for specific interactions of reading domains with modified histone peptides. To validate the method, we tested the binding specificities of seven known epigenetic reading domains on Celluspots peptide arrays, viz. the HP1ß and MPP8 Chromo domains, JMJD2A and 53BP1 Tudor domains, Dnmt3a PWWP domain, Rag2 PHD domain and BRD2 Bromo domain. In general, the binding results agreed with literature data with respect to the primary specificity of the reading domains, but in almost all cases we obtained additional new information concerning the influence of secondary modifications surrounding the target modification.

**Conclusions:**

We conclude that Celluspots peptide arrays are powerful screening tools for studying the specificity of putative reading domains binding to modified histone peptides.

## Background

Epigenetic signals include the methylation of DNA, the post-translational modification (PTM) of the N-terminal histone tails and non-coding RNAs. In eukaryotes, these epigenetic marks are involved in the regulation of gene expression and chromatin state. The most studied histone tail modifications are acetylation of Lys, methylation of Lys or Arg leading to mono-, di- (symmetric or asymmetric in the case of Arg) or trimethylation in the case of Lys and phosphorylation at Ser or Thr [1-4]. These PTMs are recognized and bound by specific reading domains which mediate most of the biological functions of histone tail PTMs [5,6]. Up to date more than 100 different PTMs have been discovered in histone tails, with many of them known to have distinct and important roles in the regulation of gene expression, DNA repair and replication, chromatin biology and the cell cycle. While histone lysine acetylation has a general activating role on transcription, histone lysine methylation can function both as an activating or a repressing mark depending on the site of methylation and the number of methyl groups added.

Acetylated histones are recognized by Bromo domains, an about 110 amino acid residues long domain folded into a left-handed four α-helical bundle. The family of Bromo domains has more than 70 identified members which are found in many chromatin-associated factors, including histone acetyltransferases or chromatin-remodeling factors. The Bromo domain containing protein 2 (BRD2) belongs to the Bromo Domain And Extra-Terminal Domain (BET) family, members of which contain two Bromo domains and an additional conserved terminal domain. It was reported that the tandem Bromo domains of BRD2 bind to H4K12ac [7].

Protein domains belonging to the Royal family include among others Tudor, Chromo and MBT domains. They are known to interact with methylated lysine residues. The Chromatin Organization Modifier (Chromo) domain is about 50 amino acids in size which are folded into a small ß-finger flanked by one α-helix. The Chromo domain family consists of more than 120 identified members. The Chromo domain of the Heterochromatin protein 1 beta (HP1ß) binds specifically to H3K9me3 and with weaker affinity to H3K9me2 and it is involved in the establishment of heterochromatin [8,9]. Another example of a Chromo domain containing protein, though less characterized, is the M-phase phosphoprotein 8 (MPP8), which has been shown to recognize H3K9me3, but also H3K9me1 and H3K9me2 [10-13].

The Tudor domain folds into a ß-sandwich flanked by one α-helix. Members of this domain family are for example the p53 binding protein 1 (53BP1), which has been shown to interact with H4K20me2 [14,15] and H3K79me2 [16], and the Jumonji domain containing protein 2A (JMJD2A) reported to bind to H4K20me3, H4K20me2, H3K4me3, H3K4me2 and H3K9me3 [14,17,18].

However, a specific interaction with modified amino acids is possible in other families as well. For example, the PWWP domain (Proline Tryptophan Tryptophan Proline Motif) present in DNA methyltransferase 3a was shown to read H3K36me3 [19] and the Plant Homeodomain (PHD) fingers, which are found in more than 100 proteins, interact with methylated lysine residues. These binding modules are about 50 amino acids long and contain two binding sites for zinc ions. The PHD finger of Rag2, an essential component of the Rag1/2 V(D)J recombinase, which mediates antigen-receptor gene assembly, interacts with H3K4me3 [20].

The investigation of the PTM specific binding of reading domains to peptides requires testing of binding to as many peptides with different PTMs as possible which is impeded by the high costs of synthetic modified peptides. Recently, we described the application of Celluspots peptide arrays for the quality assessment of commercial antibodies [21]. Peptide synthesis on cellulose membranes by the SPOT method allows the generation of many peptides with variable sequence and modifications at reasonable costs [22,23]. Peptide SPOT arrays are valuable tools for the analysis of the specificity of peptide modifying enzymes [24-28] or the binding specificity of antibodies and reading domains [19,21,22,26,28-32]. In the Celluspots technique, peptides are synthesized following the conventional SPOT synthesis on a cellulose matrix, but after the synthesis the cellulose piece together with the peptides is solubilized and spotted on glass slides [33]. Consequently, Celluspots peptide arrays are less expensive, because many arrays can be produced from one synthesis and, due to the fact that they are smaller, the assay can be performed with much less reagent.

## Results

In the present study, the binding specificities of seven known reading domains were analyzed using Celluspots peptide arrays comprising 384 peptides from 8 different regions of the N-terminal histone tails, viz. H3 1-19, 7-26, 16-35 and 26-45, H4 1-19 and 11-30, H2A 1-19 and H2B 1-19. The arrays are commercially available from Active Motif and feature 59 post-translational modifications (most of them identified, some of them hypothetical) in many different combinations (Additional file 1). Binding of the GST fused reading domain proteins to peptide arrays was visualized using an anti-GST antibody, followed by a secondary anti-goat-HRP antibody and ECL detection system. The domains were selected to represent the different folds of reading domains and show a wide range of specificities. Control experiments showed that GST alone did not give rise to any signal on the peptide array (data not shown). Each reading domain was tested at least two times on the peptide arrays to ensure that the results are reliable. In case of weak signals, the experiment was repeated with higher protein concentration. In case of an overexposed image, the protein concentration was reduced. For quality control, each glass slide contains two identical copies of the array. The binding intensities for each tested reading domain were analyzed with the Array Analyze program, which calculates the average of the binding intensities to corresponding peptide spots in both copies of the array and prepares a graphical output - one scatter plot illustrating the binding intensities observed at corresponding spots in both copies of the array and a bar diagram showing the distribution of deviations of the binding intensities to the corresponding spots. For all arrays, the main error range of the two internal duplicates was between 0 and 5% indicating that binding of reading domains to the arrays was reproducible.

### Peptide binding of the HP1ß Chromo domain

**Figure 1:**
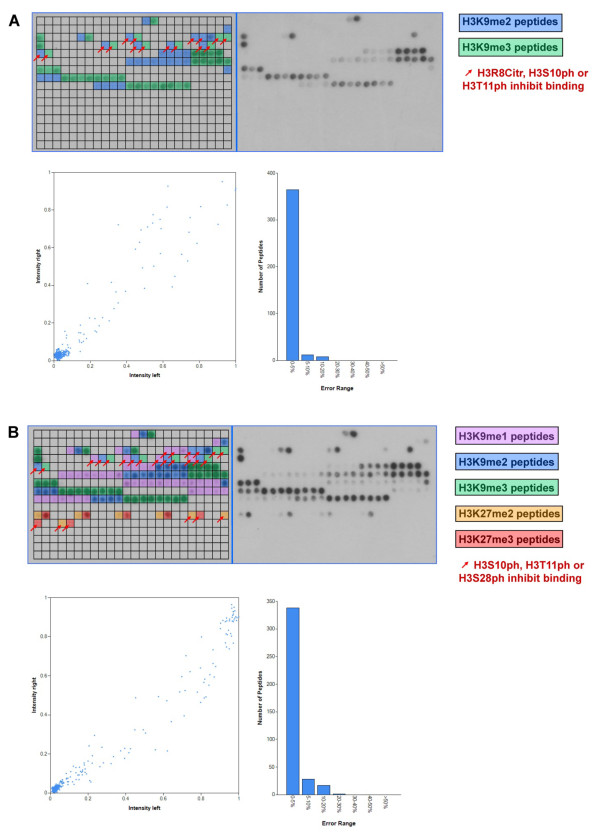
**Analysis of the binding specificity of Chromo domains on Celluspots peptide arrays**. A) Binding analysis of HP1ß. The upper part of each array analysis shows the image of the array. Peptide spots are annotated on the left copy of the duplicates. The color code is described on the right side of the image. The red arrows indicate unbound peptide spots carrying the target modifications and secondary inhibiting modifications, which are specified on the right side of the image. In the lower part of the figure, a scatter plot of the binding intensities to corresponding peptides on both identical copies of the array and a bar diagram indicating the range of deviations is shown. B) Binding analysis of the MPP8 Chromo domain. For a description of the figure see legend of panel A.

The HP1ß Chromo domain is known to recognize H3K9me3 and to a lower degree H3K9me2 [8,9]. We tested HP1ß full length protein binding to the modified histone tail peptides on Celluspots peptide arrays (Figure 1A) and observed the expected specificity towards H3K9me3/2, with a clear preference for H3K9me3. One big advantage of the Celluspots histone tail peptide arrays is that there are up to 4 different modifications on one peptide giving rise to the detailed specificity analysis of enhancing or inhibiting secondary modifications. On the array it was very clear that H3S10ph prevented binding of HP1ß to H3K9me3/2 (Figure 1A). This result is in agreement with literature, because S10P has been shown previously to prevent binding of HP1ß to H3 peptides and it has been found to release it from H3K9me3 modified chromatin in vivo [34]. Additionally we found that H3R8Citr and H3T11ph also inhibited binding of H3K9me2/3 peptides and H3R8me2s reduced binding of H3K9me2, which to our knowledge has not been reported so far. Secondary modifications like H3R8me2a/s, H3K14ac, H3R2me2a, H3K4me1/2/3 or H3K4ac had no or only a mild effect on HP1ß binding to H3K9me3/2.

**Figure 2:**
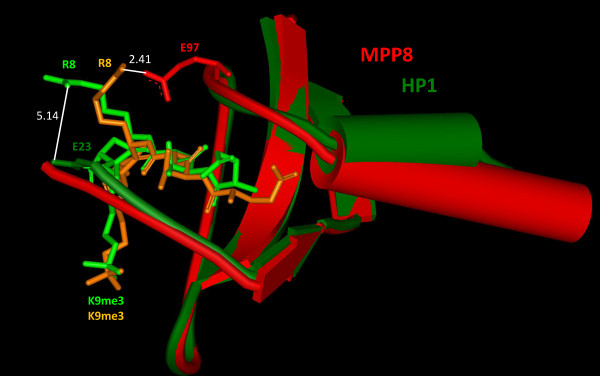
**Superposition of the HP1ß (pdb entry **1KNE**) (dark green) and MPP8 Chromo domains (pdb entry **3QO2**) (red) in complex with H3K9me3 peptides (light green for HP1 and orange for MPP8)**. 232 backbone atoms of both domains were superimposed with a root mean square of 0.92 Å using Deep View Swiss PDB viewer 3.7. The distance between the closest atoms of E23 of HP1 and R8 of the peptide is 5.14 Å. In MPP8, E97 approaches R8 more closely, with a distance of only 2.41 Å between the closest atoms.

**Figure 3:**
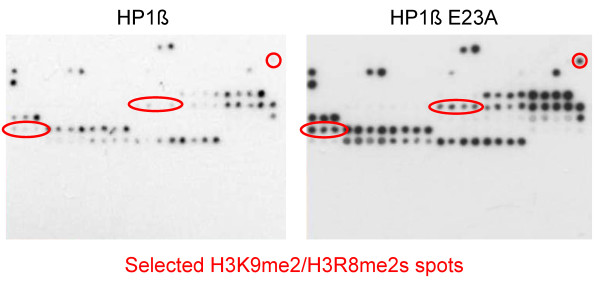
**Binding of wild type HP1ß and its E23A variant to Celluspots arrays showing similar binding specificity**. Highlighted are some of the spots which carry H3K9me2 and H3R8me2s, some of them together with other modifications. The E23A variant binds better to these spots indicating that the negative effect of H3R8me2s on binding of H3K9me2 is reduced by the E23A mutation.

In the structure of HP1ß bound to the H3K9me3 peptide (pdb entry 1KNE) [35] (Figure 2), E23 is the only residue that closely approaches R8. To study the role of E23 in R8 recognition, we generated and purified the E23A variant and studied its peptide interaction on Celluspots arrays. As shown in Figure 3, there was no general change in specificity. However several spots containing H3K9me2 combined with R8me2s, which were not bound by wild type HP1ß, were bound by the E23A variant indicating that the inhibitory effect of R8me2s was alleviated in the E23A variant. This result illustrates the application of Celluspots arrays in the specificity analysis of variants of reading domains.

### Peptide binding of the MPP8 Chromo domain

**Figure 4:**
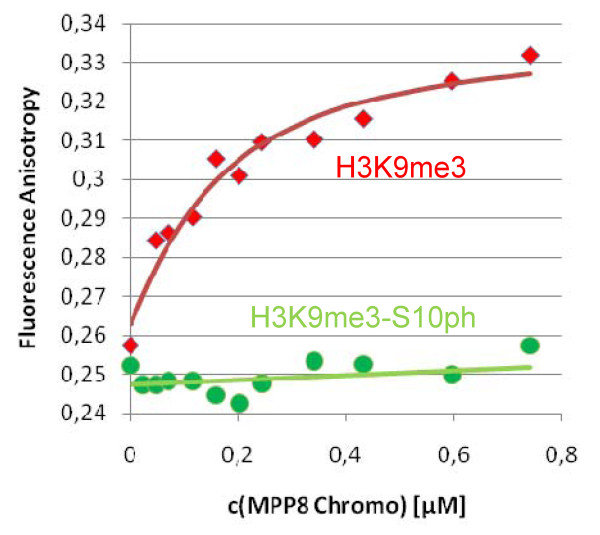
**Binding of the MPP8 Chromo domain to H3K9me3 (red) and H3K9me3-S10ph peptides (green) analyzed in solution by fluorescence depolarization**. Binding constants were determined by fitting of the data to a binary binding equilibrium to be 0.12 μM for H3K9me3 similarly as observed recently by isothermal calorimetry [13] and > 50 μM for H3K9me3-S10ph.

Next we studied the binding specificity of the MPP8 Chromo domain on Celluspots peptide arrays. The structures of the Chromo domains of HP1ß and MPP8 are similar [13] and their specificity is analogous, because the MPP8 Chromo domain is known to preferentially interact with H3K9me3, weaker with H3K9me2 and to a lesser extent with H3K9me1, but not with H3K27me3 or me2 [10-13]. On the Celluspots arrays, by far the strongest signal was observed for H3K9me3 modified peptides (Figure 1B). The secondary modifications H3R8me2a/s, H3K14ac, H3R2me2s/a, H3K4me1/2/3 or H3K4ac had very weak or no influence on peptide binding. The signal intensity for H3K9me2 binding was weak in comparison with H3K9me3, and binding to H3K9me1 only occurred if some secondary modifications were present on the peptides. As observed for HP1ß, H3S10ph or H3T11ph inhibited peptide binding. However, H3R8Citr which inhibited binding of HP1ß to H3K9me3 did not reduce binding of MPP8. In contrast to the previous studies, we observed weak binding to H3K27me3/2 as well, which was disrupted when the adjacent H3S28 was phosphorylated. Loss of binding of the H3K9me3-S10ph double modified peptide was confirmed by fluorescence depolarization measurement using purified peptides (Figure 4).

### Peptide binding of the JMJD2A double Tudor domain

**Figure 5:**
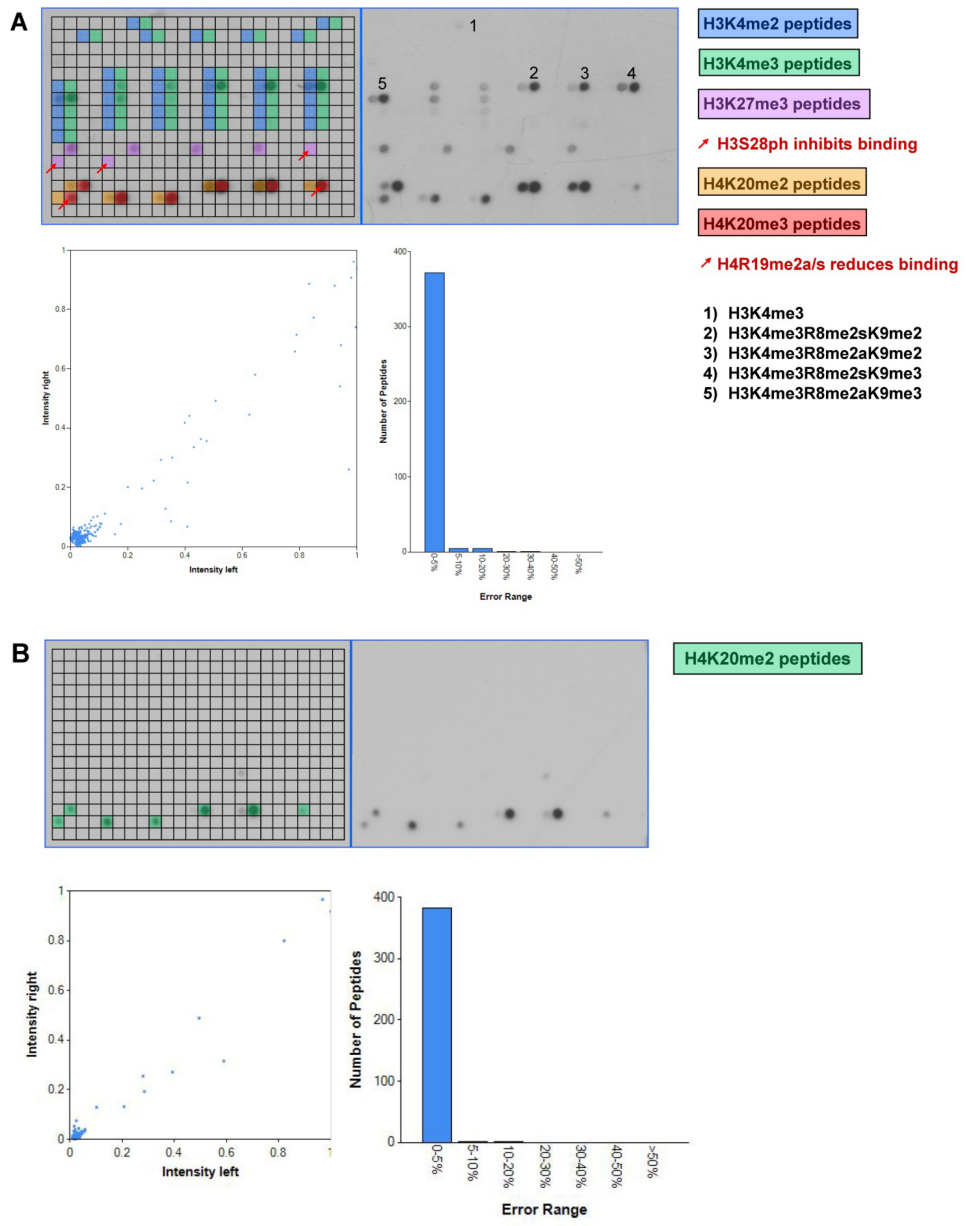
**Analysis of the binding specificity of the JMJD2A double Tudor (A) and 53BP1 tandem Tudor (B) domains on Celluspots peptide arrays**. For a description of the figure see the legend of figure 1A.

The double Tudor domain of JMJD2A was reported to interact preferentially with H4K20me3 and H4K20me2 and with weaker affinity with H3K4me3 and H3K4me2 [14,17]. Additionally, it was shown that it binds H3K9me3 with very weak affinity, which was only seen in a peptide pull-down experiment, but not on a protein microarray done in the same study [14]. On the Celluspots arrays, the strongest binding signal was observed for H4K20me3 modified peptides (Figure 5A). While the secondary modifications H4K12ac and H4K16ac had no effect on the signal intensity, asymmetric or symmetric methylation of the adjacent arginine 19 reduced binding severely. A less prominent signal reduction was observed for H4R24me2a/s as well. Following H4K20me3, H4K20me2 was the next best bound modification on the peptide array with similar effects concerning the secondary modified arginine residues at position 19 and 24. The binding to a single modified H3K4me3 was very weak (Figure 5A, spot No. 1) or in the case of H3K4me2 and H3K9me3 not detectable. However, we observed good binding (although weaker than to H4K20me3) to some peptides containing both H3K4me3 and H3K9me3 or H3K9me2 (Figure 5A, spots No. 2-5). While H3R8me2a/s did not influence binding of the double Tudor domain to these modified peptides, H3R2me2a/s abolished binding. Finally, we observed H3K27me3 binding by the double Tudor domain, which has not been reported so far. The binding signal for H3K27me3 was comparable with the intensity for H4K20me2. Phosphorylation of H3S28 as a secondary modification prevented binding of the double Tudor domain to H3K27me3. Former studies on the interaction of the JMJD2A double Tudor domain with modified histone tails did not include H3K27me3 [14], therefore this interaction should be validated by additional experiments.

### Peptide binding of the 53BP1 tandem Tudor domain

In the past, some histone modifications were reported to interact with the tandem Tudor domain of 53BP1: H3K79me2 [16], a modification which is not present on the Celluspots peptide array, and H4K20me2, H4K20me1, H3K4me2 and H3K9me2 [14,15]. Indeed, all H4K20me2 modified peptides (the main target reported for the tandem Tudor domain of 53BP1 [14]) were specifically recognized by the 53BP1 tandem Tudor domain on the peptide array (Figure 5B). Interestingly, the secondary modifications H4K16ac, H4K12ac and, to a lesser degree, H4R24me2a enhanced the binding affinity of the tandem Tudor domain for H4K20me2, since the peptides carrying H4K20me2 combined with those modifications showed the strongest binding. The other reported interactions with H4K20me1, H3K4me2 and H3K9me2 [14] were not observed on the peptide array.

### Peptide binding of the Dnmt3a PWWP domain

**Figure 6:**
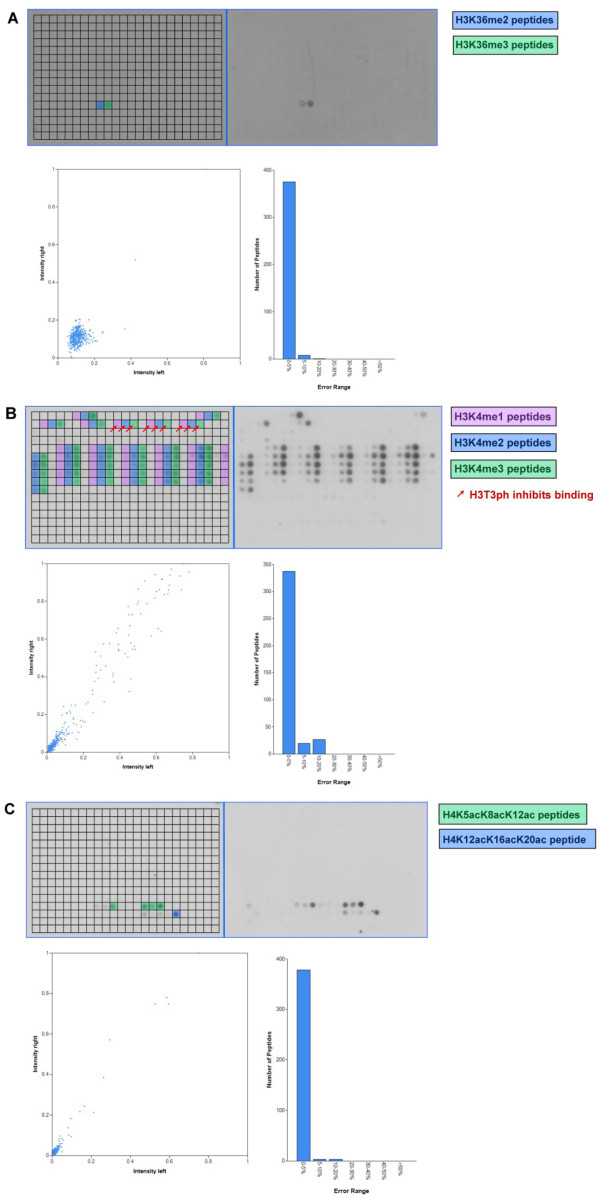
**Analysis of the binding specificity of the Dnmt3a PWWP domain (A), the Rag2 PHD finger (B) and the BRD2 second Bromo domain (C) on Celluspots peptide arrays**. For a description of the figure see legend of figure 1A.

Another reading domain which was tested on the Celluspots peptide arrays is the PWWP domain of Dnmt3a. We observed previously a specific interaction of the Dnmt3a PWWP domain with H3K36me3 on SPOT arrays, which was confirmed in follow-up experiments [19]. In agreement with this finding, the PWWP domain specifically bound to H3K36me3 modified peptides on Celluspots arrays as well (Figure 6A). Since there is only one H3K36me3-modified peptide spot, the effect of enhancing or inhibiting secondary modifications could not be studied in this case. Except for H3K36me2, which gave rise to a weaker binding signal in comparison to H3K36me3, there were no other modifications recognized by the Dnmt3a PWWP domain on the peptide array, showing a high specificity for methylated H3K36.

### Peptide binding of the Rag2 PHD finger

Out of the group of H3K4me3 interacting PHD finger binding modules, we selected the PHD finger of Rag2 for this study which has been reported to interact with H3K4me3 [20]. As expected, the strongest signal was observed for H3K4me3 modified peptides on the Celluspots arrays (Figure 6B). In comparison to H3K4me3, the signal intensity of bound H3K4me2 was greatly reduced and there was almost no binding signal observed for H3K4me1 modified peptides. In concert with literature, we found that the PHD finger of Rag2 is highly specific for H3K4me3, because there were no other modified amino acid residues targeted on the peptide array. The secondary modification H3T3ph completely abolished the binding of Rag2 PHD finger to H3K4me3-modified peptides.

### Peptide binding of the BRD2 Bromo domain

The Bromo domain protein BRD2 had been shown to interact with H4K12ac-modified chromatin [7] and the second Bromo domain of BRD2 was found to recognize H4K5K12-diacetylated peptides [36,37]. Therefore, we tested the second Bromo domain of BRD2 on Celluspots peptide arrays and found that it bound preferentially to tri- or tetra-acetylated peptides from histone H4 (Figure 6C) with some preference for H4K5acK12ac. The tetraacetylated peptide H4K5ac-K8ac-K12ac-K16ac showed the strongest binding signal. The hypothetical modification H4K20ac is included on the peptide array and the triacetylated H4K12ac-K16ac-K20ac peptide was recognized by the Bromo domain with similar affinity as the other triacetylated H4 peptides. Notably the monoacetylated peptides H4K5ac, H4K8ac, H4K12ac and H4K16ac were not bound and diacetylated peptides containing H4K5ac-K8ac were only weakly bound. This is not surprising, since it was shown in the past that some Bromo domains preferentially bind to multiple acetylated histone tails [38].

## Discussion

Reading domains mediate PTM specific protein/protein interactions, in the case of epigenetic reading domains, a PTM specific interaction with histone peptides occurs. These protein domains are essential players in epigenetic signaling, because they translate the specific PTM patterns of histones into a biological function. Identification and study of reading domains includes the analysis of their specificity with respect to the primary PTM recognized, the peptide sequence and the influence of additional secondary PTMs nearby. One example for a screening system for the identification of PTM binding proteins is a protein microarray used by Kim *et al. *in 2006. For that study domains of known chromatin-associated proteins were cloned as GST fusions and spotted onto nitrocellulose-coated glass slides and incubated with fluorophore-labeled N-terminal histone H3 and H4 peptides carrying different modifications [14]. Peptide arrays have been used as an alternative screening tool as well. Bua *et al. *applied peptide arrays containing biotinylated histone peptides, which were either unmodified or carried a single modification at known PTM sites [10], later larger peptide arrays also containing combinations of PTMs were used [21,31,39,40]. We applied Celluspots arrays for the screening of antibody binding to modified histone tails [21], because they allow for a cost effective presentation of many potential targets with different modification patterns.

Recently, we also used Celluspots peptide arrays for the initial screening of the binding specificity of two PHD finger like domains - the ADD domains of ATRX and Dnmt3a [31,40]. The ADD domain of Dnmt3a was reported to bind to unmodified H3K4 and the structure of this complex had been solved [41]. On the peptide array, the Dnmt3a ADD domain interacted only with peptides where H3K4 is either unmodified or monomethylated, but not when it is di- or trimethylated [31]. While secondary modifications like H3R2me2a/s had no or only a mild effect on the binding affinity, H3T3P, H3S10ph and H3T11ph prevented binding of the Dnmt3a ADD domain. We have shown that the ATRX ADD domain binds to H3K9me3 in the absence of H3K4me2/3 on the peptide array [40] and confirmed this result using purified peptides. Later, additional experiments confirmed this finding [42,43].

Here, we tested the binding of several reading domains to Celluspots peptide arrays and show that the binding specificities observed with Celluspots arrays in general agree nicely with literature results. One of the big advantages of this approach is that many different modified peptides are presented on the array such that no initial hypothesis on the binding motif is necessary. In addition, peptides with up to four combined modifications are present, which allows for analysis of combinatorial readout to identify secondary modifications which enhance or reduce the binding affinity to peptides which carry the primary target modification.

An inhibiting effect of some secondary modifications was seen for most of the studied reading domains. For example, HP1ß binding to H3K9me3 was prevented by H3R8Citr, H3S10ph and H3T11ph. All of these modified amino acids are either close or adjacent to the target trimethyl lysine, but an additional modification at an adjacent residue does not necessarily influence binding as seen in the case of the MPP8 Chromo domain. Even though binding was inhibited by H3S10ph and H3T11ph similarly as for HP1ß, H3R8Citr did not have any effect on MPP8 Chromo domain binding to H3K9me3. Trying to understand that difference, we superimposed the structures of HP1ß (pdb entry 1KNE) [35] and MPP8 Chromo domain (pdb entry 3QO2) [13] in complex with H3K9me3 peptides and compared the distances of unmodified R8 in the peptides to the nearest side chain atoms of the Chromo domains, which are E23 in HP1ß and E97 in MPP8 (Figure 2). In MPP8, the distance between the E97 side chain atoms and R8 is 2.41 Å indicative of a strong hydrogen bond being formed, that also would be present after citrullination of the arginine. In contrast, in HP1ß the nearest side chain to R8 is E23 with a distance of 5.14 Å, which may provide some electrostatic interaction but does not support a hydrogen bond. The electrostatic contact between E23 and R8 would be lost after citrullination, because citrullination of arginine removes its charge which may explain why citrullination of H3R8 prevents binding of HP1ß but not of MPP8.

We observed with several domains that the presence of one or more additional modifications improved binding to peptides which carried the primary mark. This effect could be due to technical problems like unequal peptide synthesis or surface binding. It could also mean that these combinations of PTMs are biologically important, like in the case of HP1ß only binding to H3K9me3 if S10 is not phosphorylated [34] or the ATRX ADD domain only binding to H3K9me3 if K4 is unmethylated [40]. Furthermore, one may also speculate that improved binding by the presence of additional PTMs may indicate that the amino acid sequence of the peptides used is not ideal for binding of that reading domain, which would suggest binding to other modified non-histone proteins. Therefore, the biological relevance of enhancing or inhibiting secondary modifications found in an initial screening for specific interactions of a reading domain with modified peptides needs to be further investigated with additional experiments. In the case of HP1ß, for example, it has been shown that phosphorylation of H3S10 during the M-phase of the cell cycle leads to the release of HP1 proteins from H3K9me3 modified chromatin [34] such that this detail of the array results has a biological meaning.

**Figure 7:**
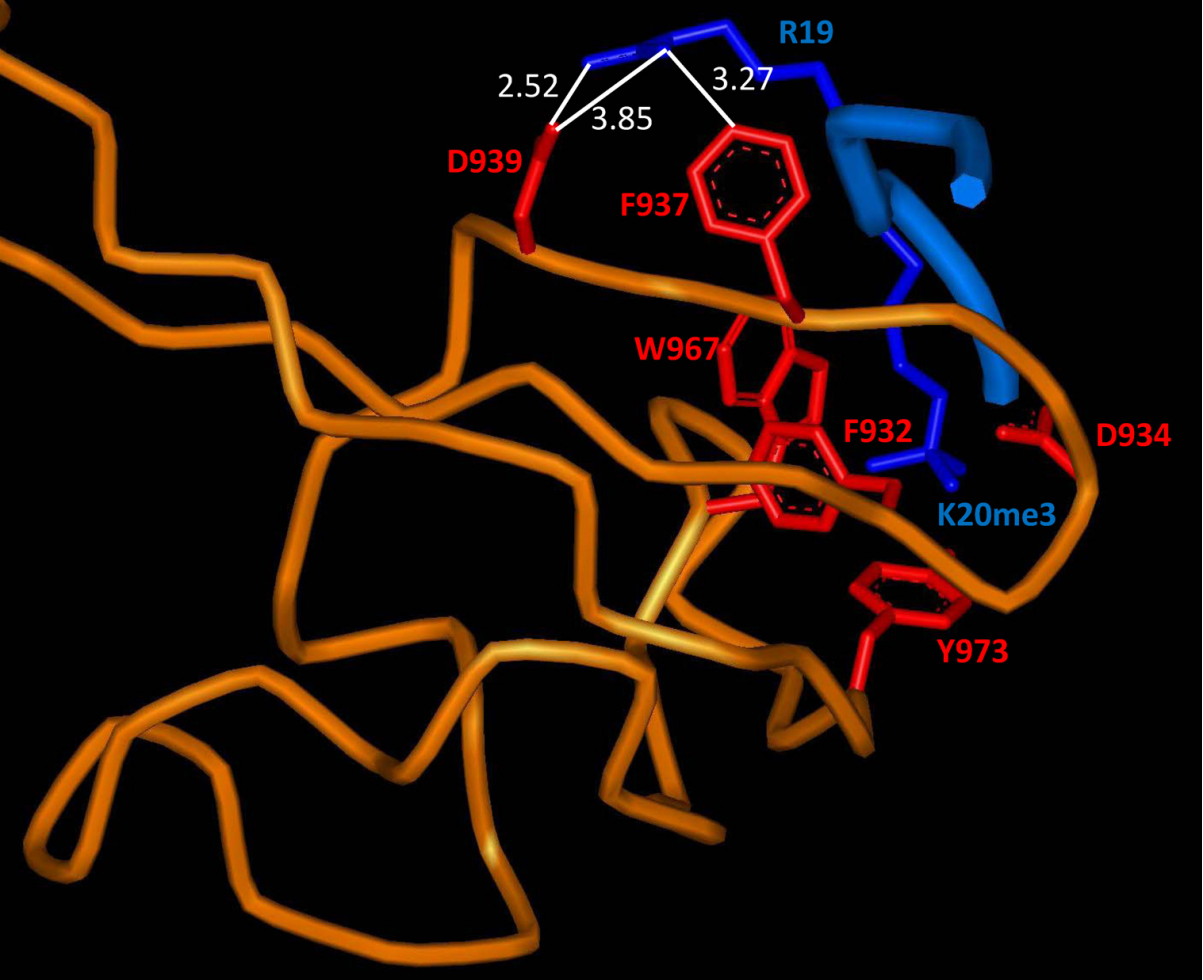
**Interaction between JMJD2A double Tudor domain and R19 in H4K20me3 peptide**. The backbone of JMJD2A double Tudor domain (pdb entry 2QQS) is shown in orange. Amino acid side chains of the Tudor domains either interacting with H4K20me3 or H4R19 are shown in red. The H4-peptide backbone is shown in a medium blue with the side chains of K20me3 and R19 highlighted in a dark blue. The side chains of F932, D934, Y973 and W967 form an aromatic cage around the trimethylated lysine 20, while the side chains of F937 and D939 interact with unmodified R19. The distances of the side chains atoms of D939 and F937 to R19 are indicated.

Another example of the inhibition of binding by a secondary modification was seen with the JMJD2A double Tudor domain binding to H4K20me3. While H4K12ac and H4K16ac had no effect on the signal intensity, we observed that asymmetric or symmetric methylation of the arginine 19 reduced binding of the JMJD2A double Tudor domain to H4K20me3 severely. This observation can be explained in the light of the crystal structure of the JMJD2A double Tudor domain in complex with the H4K20me3 peptide (pdb entry 2QQS) [18] (Figure 7). The double Tudor domain amino acid side chains of D939 and F937 are in close proximity to the unmodified arginine 19 of the H4 peptide. On the basis of this, we speculate that the so far hypothetical methylation of arginine 19 would interfere sterically with the positioning of D939 and/or F937, which may explain the reduced binding of the double Tudor domain of H4K20me3/H4R19me2a/s double modified peptides observed on the peptide array.

As described, with the JMJD2A double Tudor domain, we observed combined readout of H3K4me3 and H3K9me3 which is interesting, because both marks have opposing biological effects. Since JMJD2A is known to demethylate H3K9me3 [44,45], one could speculate that H3K4me3/K9me3 dual modified chromatin is an intermediate in the reactivation of H3K9me3 silenced chromatin, where trimethylation of K4 would recruit the JMJD2A activity that would finalize the switch from H3K9me3 repressed to H3K4me3 active chromatin. Interestingly, the ATRX ADD domain performs a combined readout of H3K4 and K9 as well, but in this case the preferred combination is H3K4me0 and H3K9me3, which is both characteristic of transcriptionally inactive chromatin.

## Conclusions

We describe the application of Celluspots peptide arrays which contain 384 histone peptides carrying 59 post-translational modifications in different combinations as an inexpensive, reliable and fast method for initial screening for specific interactions of reading domains with modified histone peptides. Since peptide arrays are screening tools, unexpected or novel results need to be confirmed by equilibrium peptide binding experiments using purified peptides. In our experience, such studies often confirmed results from peptide arrays. For example in the case of the Dnmt3a PWWP domain, binding to H3K36me3 on the peptide array could be verified by peptide binding, pull-down of native nucleosomes and functional DNA methylation experiments [19]. Similarly, the initial observation of a combinatorial readout of H3K9me3 when H3K4 is not di- or trimethylated by the ATRX ADD domain on the peptide array was confirmed by chromatin pull-down and peptide binding assays in our laboratory [40] and later also by others [42,43]. The same is true for the Dnmt3a ADD domain recognition of unmodified H3K4, which is important for the methylation of DNA by Dnmt3a, where peptide array results [31] nicely agreed with published equilibrium peptide binding data [41]. Here, we confirmed by peptide binding that MPP8 Chromo domain binding to H3K9me3 is inhibited by S10ph. In all these cases, the initial peptide array results prompted further experiments, which confirmed them and in some cases it was possible to show a biological relevance. We conclude that Celluspots peptide arrays are well suited tools to study the PTM specific interactions of reading domains and reading domain variants with modified histone tails.

## Methods

### Cloning, expression and purification of reading domains

**Figure 8:**
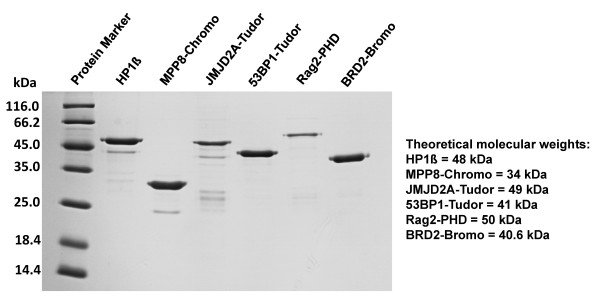
**Coomassie stained SDS gel showing the purified proteins used for peptide binding experiments**. Loading was not adjusted to protein concentrations. The purification of the Dnmt3a PWWP domain is shown in Dhayalan et al. (2010) [19].

The sequences encoding human HP1ß full length (residues 1-185), the Chromo domain of human MPP8 (residues 58-112), the double Tudor domain of human JMJD2A (residues 856-1047), the tandem Tudor domain of human 53BP1 (residues 1490-1608), the PHD finger of human Rag2 (residues 311-520) and the second Bromo domain of human BRD2 (residues 348-455) were amplified from cDNA derived from HEK293 cells and cloned as GST fusion proteins into the pGEX-6P-2 vector (GE Healthcare). The sequence encoding the PWWP domain of murine Dnmt3a (residues 279-420) was subcloned as GST-fusion protein into the pGEX-6P-2 vector (GE Healthcare). The GST-tagged proteins were expressed and purified as described [28] (Figure 8). Protein concentration was determined by UV spectroscopy. Site directed mutagenesis was performed as described [40].

### Peptide arrays

Celluspots peptide arrays spotted on glass slides were provided from Intavis AG (Köln, Germany). They are now commercially available from Active Motif (Cat. No. 13001). The peptide sequences and PTMs are specified in the Additional File 1. For quality control, each glass slide contains two copies of the array. We have shown previously that antibody binding to these internal duplicates was highly reproducible which ensures reproducible peptide spotting [21]. Similar results were observed here with reading domains. By mass spectrometric analysis, we showed previously that the peptide spots contained the full length product and sometimes some shorter by-products as well [21] which is expected, since unpurified peptides are used. These heterogeneous contaminating peptides did not affect antibody binding in a detectable manner, because the arrays present much more peptides than available surface binding sites for antibodies or reading domain. Antibody binding to arrays prepared from independent peptide synthesis was highly reproducible [21].

### Binding of protein domains to peptide arrays

The array was blocked by incubation in TTBS buffer (10 mM Tris/HCl pH 7.5, 0.05% Tween-20 and 150 mM NaCl) containing 5% non-fat dried milk at 4°C overnight, then washed two times with TTBS buffer, one time with interaction buffer (100 mM KCl, 20 mM HEPES pH 7.5, 1 mM EDTA, 0.1 mM DTT and 10% glycerol), and incubated with purified GST-tagged reading domains: HP1ß full length protein (10 nM), MPP8 Chromo domain (0.5 μM), JMJD2A double Tudor domains (10 nM), 53BP1 tandem Tudor domain (1 μM), Dnmt3a PWWP domain (50 nM), Rag2 PHD finger (2 nM) and BRD2 Bromo domain 2 (10 nM) at room temperature for 2 hours in interaction buffer. After washing with TTBS buffer three times, the array was incubated with goat anti-GST antibody (GE Healthcare #27- 4577-01) 1:5000 dilution in TTBS buffer containing 1% non-fat dried milk for 1 h at room temperature. Then, the membrane was washed three times with TTBS and incubated with horseradish peroxidase conjugated anti-goat antibody (Invitrogen #81-1620) 1:12000 in TTBS containing 1% non-fat dried milk for 1 h at room temperature. Finally, the membrane was washed four times with TTBS and submerged in ECL developing solution (Thermo Fisher Scientific) and image was captured on an X-ray film. Typical exposure times were 0.5-5 min. Analysis was done using the Array Analyze program, which was also used to prepare the graphs shown in Figures 1, 5 and 6. The program runs under MS-Windows and it is available free of charge at http://www.activemotif.com/catalog/667.html or from the authors upon request.

### Peptide binding experiments

Peptide binding of the MPP8 Chromo domain was analyzed by fluorescence depolarization using a Varian Carry Eclipse fluorescence spectrophotometer as described [40]. Purified FITC-coupled peptides (H3K9me3 and H3K9me3-S10ph, both amino acids 1-19) were purchased from Intavis AG (Köln, Germany).

## Authors' contributions

IB, AD, SK, GK and RT conducted and analyzed the experiments. IB, AD and AJ designed the study. IB and AJ were involved in data analysis and interpretation and drafted the manuscript. All authors read and approved the final manuscript.

## Supplementary Material

Additional file 1 **Supplemental information S1.xls**. Compilation of the peptides presented on the Celluspots arrays. Excel file showing the sequences, modifications and positions of all peptides on the Celluspots arrays. Click here for file
